# Integrative Group Medical Visits: A National Scoping Survey of Safety-Net Clinics

**DOI:** 10.1089/heq.2018.0081

**Published:** 2019-01-25

**Authors:** Ariana Thompson-Lastad, Paula Gardiner, Maria T. Chao

**Affiliations:** ^1^ Osher Center for Integrative Medicine, UC San Francisco, San Francisco, California.; ^2^ Department of Family Medicine, University of Massachusetts Medical School, Worcester, Massachusetts.; ^3^ Division of General Internal Medicine and Osher Center for Integrative Medicine, UC San Francisco, San Francisco, California.

**Keywords:** integrative medicine, community health centers, safety-net providers, chronic disease

## Abstract

**Purpose:** Integrative group medical visits (IGMVs) aim to increase access to complementary and integrative health care, which is particularly relevant for low-income people. We sought to describe IGMV programs in US safety-net clinics through a survey of providers.

**Methods:** An online and paper survey was conducted to collect data on the use of complementary health approaches and characteristics of IGMV programs. We recruited a purposive sample of safety-net clinicians via national meetings and listservs.

**Results:** Fifty-seven clinicians reported on group medical visits. Forty percent worked in federally qualified health centers, 57% in safety-net or teaching hospitals, 23% in other settings such as free clinics. Thirty-seven respondents in 11 states provided care in IGMVs, most commonly for chronic pain and diabetes. Nutrition (70%), mindfulness/meditation/breathing (59%), and tai chi/yoga/other movement practices (51%) were the most common treatment approaches in IGMVs.

**Conclusion:** Safety-net institutions in 11 states offered IGMVs to treat a range of chronic conditions. IGMVs are an innovative model to improve access to non-pharmacologic approaches to chronic illness care and health promotion. They may advance health equity by serving patients negatively impacted by health and health care disparities.

## Introduction

Over one-third of adults in the United States use complementary health approaches, most commonly for chronic disease management.^1,2^ As defined by the National Institutes of Health, “complementary health approaches” include natural products (e.g., herbs, vitamins) and mind–body practices such as acupuncture and meditation.^2–4^ Use is lower among those who are publicly insured (25%), uninsured (23%), or living in poverty (21%).^2^ Integrative health care, which combines biomedical care with complementary health approaches,^5^ may improve quality of care by supporting patient preferences and increasing access to non-pharmacological treatment for conditions including chronic pain and diabetes.^6^ However, complementary and integrative care is generally less accessible to uninsured and Medicaid/Medicare patients due to out-of-pocket cost.^7,8^

Group medical visits (GMVs), or shared medical appointments,^9^ are widespread in US primary care and growing in use across medical specialties. GMVs bring 5–20 patients to the same space for medical care, health education, and peer support. Providers bill patients' insurance as for a standard medical appointment and spend 1–3 h with a group of patients. GMVs are commonly used for prenatal care^10^ and diabetes^11^ and increasingly for chronic pain to support safe use of opioids,^12^ access to medication-assisted treatment,^13^ and availability of non-pharmacological treatment.^14,15^ Research indicates that GMVs are associated with comparable or better health outcomes than individual care.^11,16^ GMVs may also decrease health care costs, in part by reducing emergency room visits.^17–19^

In the past decade, some safety-net clinics have begun offering integrative GMVs (IGMVs). These combine GMVs' core elements of peer support, health education, and biomedical care with access to complementary health approaches such as yoga, acupuncture, or meditation. Small studies suggest IGMVs are a promising approach for chronic health conditions and health promotion more broadly, with positive effects on physical and mental health. IGMVs for chronic pain have been associated with lower pain intensity and opioid medication use,^15^ improved health-related quality of life and sleep,^20–23^ and reduced depressive symptoms and loneliness.^24^ Existing research suggests that stress reduction via increased empowerment in IGMVs potentially contributes to improved health outcomes.^25^

Although IGMVs are a growing trend, little is known about where and how broadly they are being implemented. Small clinical pilots have provided data on individual-level outcomes in IGMVs. However, scant information is available on safety-net programs not funded as research. Given the prevalence of GMVs in safety-net settings and the growth of integrative care nationally, we hypothesized that GMVs would be present in regions throughout the United States. Our scoping survey sought to describe the structure and scope of care being provided in safety-net IGMVs. We sought out a range of safety-net IGMV programs, examining which health conditions are treated in IGMVs, which complementary health approaches are most common, and what providers view as successful and challenging aspects of this emerging approach to care.

## Methods

### Study design

This survey is part of a larger, mixed-methods study of IGMVs in safety-net clinics. The study included a national survey, qualitative patient and staff interviews, and ethnographic observations at four community health organizations. Qualitative data (reported separately) aimed to provide in-depth understanding of well-developed safety-net IGMV programs.^26^ The survey was developed by the authors in consultation with clinicians with IGMV experience. We included questions on the scope and structure of IGMVs around the United States. To assess heterogeneity of IGMVs, we asked for information about a wide range of complementary health approaches, including some that are common in biomedical care (e.g., nutrition counseling), others that are increasingly viewed as evidence-based care (e.g., acupuncture), and still others that remain more controversial (e.g., homeopathy).

### Sample

We distributed the survey at 2016 professional meetings of the Academic Consortium for Integrative Medicine and Health, and Integrative Medicine for the Underserved. We sent electronic invitations to complete the survey through: (1) listservs of Indian Health Service and Integrative Medicine for the Underserved; (2) social media sites of professional networks of providers involved with safety-net care; and (3) ∼90 clinics whose websites stated that they provide care in IGMVs. Additional respondents were recruited through snowball sampling. We chose a purposive, non-probability sampling approach to gather data from a targeted sample of providers with specific expertise in IGMV practice.

### Inclusion/exclusion criteria

Eligibility was limited to health care providers who were (1) trained in biomedicine and/or complementary health approaches, and (2) provided care in GMVs, which we defined in the survey as “medical care provided to multiple patients in the same room, when insurance is billed for at least some of these patients.” Some questions were answered by all respondents. Others were answered only by those respondents providing care in IGMVs, defined as including all of the following:

- Care provided to multiple patients in the same room
- Visit billed using ICD-10 codes and documented in patients' medical records
- At least one complementary health approach (e.g., acupuncture, mindfulness, yoga) incorporated in most group sessions and
- Patients interact with each other during the group session.

### Data collection and informed consent

An English-language questionnaire with open- and closed-ended items was developed by the authors and entered into Qualtrics, a secure survey management tool. Questions included respondents' demographic characteristics (e.g., ethnicity, age); information about their workplaces (e.g., location, kinds of insurance accepted); and details about group visit and integrative health programs at their workplaces (e.g., conditions treated in integrative group visits, complementary health approaches offered). Open-ended questions elicited respondents' favorite aspects of integrative group visits, greatest challenges, and what they wanted to learn about similar programs at other organizations. Potential respondents accessed the web-based survey through Qualtrics and provided informed consent before continuing. For surveys administered at conferences, respondents completed a paper consent form before filling out the survey. The UC San Francisco institutional review board approved all study procedures.

### Data analysis

Data from paper surveys were entered into Qualtrics; web-based surveys were completed in Qualtrics. Of 61 completed surveys, we identified four cases from multiple staff of a single organization; in these cases, we used the first respondent's data and removed additional respondents' data from analysis, yielding a final sample of 57 surveys. We calculated descriptive statistics including mean, median, and standard deviations using SPSS version 24. We analyzed qualitative data using thematic analysis.^27^ Two authors (A. T.-L. and P.G.) independently coded the qualitative responses, completed by 31 respondents, then discussed coding and agreed on primary themes.

## Results

### Demographics

Respondents had a mean age of 50 and were primarily female (90%) and White (83%), with some providers from other ethnic groups (11% Hispanic/Latino, 7% Asian or Pacific Islander, and 9% other race/ethnicity; see Table 1). Providers had an average of 6 years of experience with GMVs. Forty-two percent were physicians; 16% were nurse-practitioners, nurse-midwives, or physician assistants; and 16% were mental health care providers. Many identified themselves as having multiple professional roles, for example, physician and yoga teacher.

**Table 1.** **Characteristics of Participants and Workplaces (*N*=57)**

Characteristics	*N* (%)
Age in years±standard deviation	50±10
Gender	
Male	6 (10)
Female	51 (90)
Race/ethnicity	
White	47 (83)
Hispanic/Latino	6 (11)
Asian or Pacific Islander	4 (7)
Other (including African American, Native American)	5 (9)
Professional role^a^	
Physician (MD or DO)	24 (42)
Nurse-practitioner, physician assistant, or nurse-midwife	9 (16)
Mental health provider (psychologist, licensed social worker)	9 (16)
Acupuncturist	5 (9)
Other (including yoga teacher, group program coordinator, herbalist)	20 (35)
Average years of experience with group visits±standard deviation	5.9±6.3
Workplace	
Federally qualified health center	23 (40)
Teaching hospital/clinic	26 (46)
Safety-net hospital	6 (11)
Other (including free clinic, Indian Health Service, private practice)	13 (23)
State	
California	20 (35)
Massachusetts	5 (9)
Ohio	5 (9)
Oregon	4 (9)
Other^b^	23 (40)
Geographic area	
Urban	36 (63)
Suburban or small city	13 (23)
Rural	3 (5)
Types of insurance accepted	
Medicaid	42 (74)
Free or discounted care for uninsured	30 (53)
Medicare	41 (72)
Private insurance	37 (65)
Veteran's benefits	6 (11)
Integrative medicine services offered outside of groups (e.g., acupuncture, meditation)	51 (89)
Other group programs offered	
Therapeutic movement (e.g., yoga, tai chi)	23 (40)
Group therapy or mental health support	23 (40)
Physical activity classes	17 (30)
Peer support	15 (26)
Cooking classes	16 (28)
Substance abuse treatment	11 (19)
Arts or activity groups	9 (16)

^a^ Respondents could select multiple options; total may be more than 100%.

^b^ Other states include MN, NY, FL, WI, CO, NE, MI, PA, KY, IL, TN, NM, TX, WA, Washington DC.

### Characteristics of respondents' workplaces

**FIG. 1.**
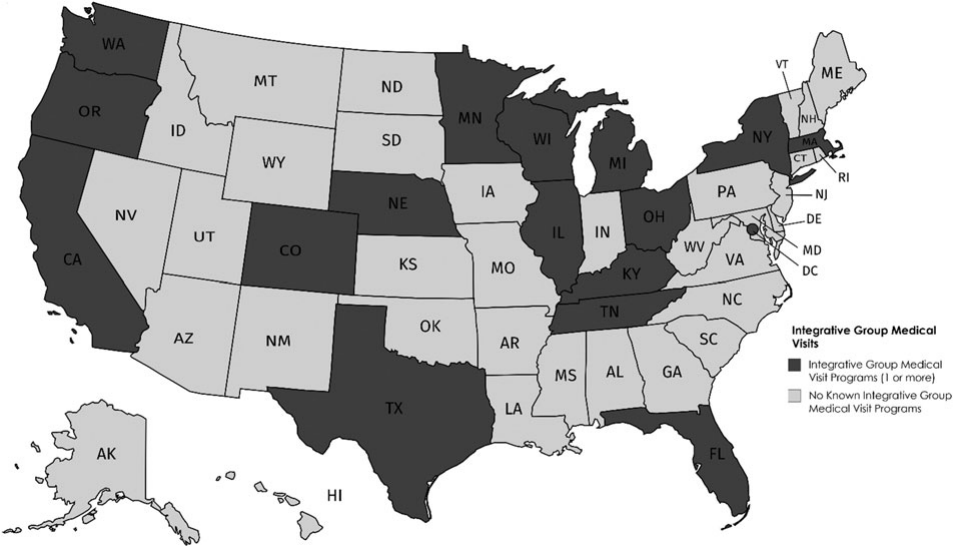
Geographic distribution of integrative group medical visits.

Forty percent of respondents worked in federally qualified health centers, 57% in safety-net or teaching hospitals, and 23% in other settings such as the VA or free clinics (Table 1). Many respondents (35%) worked in California (Fig. 1), with others in a total of 17 states including Massachusetts, Ohio, and Oregon (each 9%). The majority of providers (63%) worked in urban areas. Nearly all worked in settings that accepted public insurance, including Medicaid (74%), Medicare (72%), and/or veterans' benefits (11%), and many sites provided free or discounted care for uninsured people (53%). A majority of respondents (89%) reported that their workplaces offered integrative health care services outside of IGMVs.

### Group education and support programs

Most clinical sites also offered non-medical group education or support programs (Table 1). Most common were therapeutic movement classes such as yoga or tai chi, and group therapy or mental health support groups (each 40%). Exercise classes such as Zumba were also common (30%), as were cooking classes (28%).

### Complementary health approaches in group visits

Clinicians at 37 of the 57 sites offered care in IGMVs. The following results are based on this subsample. At the 37 IGMV sites, providers reported a wide variety of complementary health approaches (Table 2). Nutrition (70%) and mindfulness, meditation, and breathing exercises (59%) were most commonly included in IGMVs. Tai chi, yoga, or other movement practices (51%); acupuncture (46%); herbs and supplements (43%); and chiropractic, massage, or osteopathic manipulation treatment (30%) were also offered.

**Table 2.** **Characteristics of Integrative Group Medical Visits (*n*=37)**

Complementary health approaches offered in IGMV^a^	*N* (%)
Nutrition	26 (70)
Mindfulness, meditation, or breathing	22 (59)
Tai chi, yoga, or other movement	19 (51)
Acupuncture	17 (46)
Herbs or supplements	16 (43)
Chiropractic, massage, or osteopathic manipulation treatment	11 (30)
Conditions treated in IGMV	
Chronic pain	28 (76)
Diabetes	23 (62)
Cardiovascular disease or metabolic syndrome	14 (38)
Cancer	8 (22)
Mental health and/or substance use	7 (19)
Prenatal care	8 (22)
Pediatrics	6 (16)
IGMV languages offered	
Spanish	15 (40)
Korean	1 (2)
Chinese	1 (2)
Estimated number of patients attending IGMV, mean (range)	7.5 (4–15)
Frequency of IGMV sessions	
Weekly	57%
Every other week or twice a month	8%
Monthly	26%
Other	8%
Number of IGMV sessions patients are eligible to attend	
2–5	26%
6–10	29%
>10	7%
Ongoing/indefinite	38%

^a^ Respondents could select multiple options; total may be more than 100%.

IGMV, integrative group medical visit.

### Conditions treated in IGMVs

Most sites offered IGMVs to treat chronic conditions, including chronic pain (76%), diabetes (62%), and cardiovascular disease or metabolic syndrome (38%). A small number of sites treated substance use and/or mental health (19%) in IGMVs.

### IGMV program characteristics

Typical attendance in IGMVs ranged from 4 to 15 patients, with an average of 7.5 patients per session (Table 2). IGMV programs were structured in a variety of ways; 57% met weekly, 26% met monthly. In over one-third of IGMV programs (38%), patients were eligible to attend ongoing groups indefinitely; the remainder limited attendance to a set number of sessions. Some sites offered IGMVs in languages other than English, including 40% in Spanish, one program in Chinese, and another in Korean.

### Successes and challenges of IGMV programs

Providers shared qualitative responses on their favorite aspects of IGMVs, the most challenging aspects, and what they wanted to learn about programs at other organizations (Table 3). They generally reported positive experiences with IGMVs and saw benefits to both patients and clinicians participating in this model of care. Cross-cutting themes included (1) patient-related factors such as recruitment and retention; (2) staff-related factors such as how to staff and bill for the integrative aspect of IGMVs; (3) IMGV program implementation and sustainability.

**Table 3.** **Qualitative Themes and Participant Quotes**

**Most challenging aspects**	**Patients missing appointments:** “When patients are ill or they have transportation or health challenges and they miss a visit, it affects the whole group and the group dynamics.”	**Finding and paying for staff trained in complementary health approaches:** “What has been most challenging is to train our staff in the integrative modalities. We have rarely had the financial resources to hire others into the system with the expertise. Nor had the system been willing to pay for training in integrative modalities.”	**Program sustainability:** “maintaining appropriate administration/human resources support” “recruitment and programs sustainability, i.e., nursing and front office support.”
**Favorite aspects**	**Patients supporting each other and sharing expertise:** “The connection it generates for patients that would usually be isolated.” “Witnessing peer to peer learning.”	**Positive changes to patient–provider relationships:** “How the (power) dynamic between patient and provider is dissolved. Happier patients and happier providers.” “I enjoy working as a provider with a group—different dynamics than 1:1 with patients.”	**Seeing patients' health improve, integrating complementary health approaches:** “Patients actually get better and are able to significantly increase the quality of their lives as well as often diminish the pain they are experiencing. I could never get these results in a 1:1 traditional western medicine format of a doctor-patient visit.”
**Want to learn from other programs**	**How to recruit and retain patients:** “How to manage enrollment and retention in [a safety-net] population with many barriers to care.”	**Staffing and billing for group visits:** “How to serve patients with high co-pays.” “How to bill, who can bill.” “Is there a limit to how often [patients] can come and be billed for [group visits]?”	**How to measure outcomes:** “I would love to see a collective of people gathering data on these groups together, from all their different sites.” “…Interested to know what modalities were the most well received among the different populations.”

Providers noted how IGMVs allowed patients to share expertise and support one another, which several described as patient-empowering. Providers' favorite aspects of IGMVs included positive changes in patient–provider relationships. They also noted improvements in patients' physical and mental health, which they attributed to both complementary health approaches and peer support.

Commonly reported barriers included patient recruitment and retention. Specifically, respondents emphasized the need for adequate staffing and institutional support for patient recruitment, such as staff to make reminder phone calls to patients and to open facilities during evening hours when more patients are available. In addition, providers highlighted structural challenges, such as access to reliable transportation, which make it challenging for patients to participate in IGMVs. These challenges are also common in safety-net settings outside of IGMV programs. Specific challenges of IGMVs included finding and paying staff trained in integrative care given the lack of reimbursement for complementary health approaches, as well as finding ways to successfully integrate complementary health approaches in group settings.

When asked what they wanted to know about how other organizations implemented IGMVs, approaches to recruitment and retention of patients was a dominant theme. Providers also had specific questions about staffing IGMVs with appropriately trained clinicians and support staff, and implementing and billing for complementary health approaches. Several providers expressed interest in working with others in similar programs to collect data, develop best practices, and measure health outcomes of IGMVs.

We found that IGMV implementation is complicated in ways that are consistent with existing literature on GMV program implementation and safety-net care more broadly.^16,28,29^

Clinicians reported that challenges to starting and sustaining group visit programs include obtaining adequate support from their organizations and health care systems. For example, IGMVs require clinicians who are capable of facilitating a group-based care model as well as using or teaching complementary health approaches. In addition, clinic staff need time to recruit patients, develop curricula, and complete other administrative tasks to support IGMV programs.

## Discussion

In the United States, people with lower income and education levels are less likely to use complementary health approaches, despite the growing evidence base on their usefulness for chronic conditions.^2^ Our research describes an emerging model that can increase access to complementary health approaches, particularly in safety-net settings. We found that IGMVs are geographically dispersed throughout the United States. Although the number of clinics offering IGMVs remains unclear given our non-representative sample, this trend is parallel with national growth of complementary and integrative health services in settings ranging from large academic medical centers^30^ to a national network of community acupuncture clinics.^31^

We found that clinicians from a range of professional backgrounds are providing GMVs and IMGVs in safety-net settings that serve uninsured and publicly insured patients. These models offer integrative health care that low-income people struggle to afford when it requires out-of-pocket payment. Our findings indicate considerable interest in and enthusiasm for this model of care among clinicians around the country and demonstrate that IGMVs include a wide range of complementary health approaches to treat patients with a variety of chronic conditions, including diabetes and chronic pain. Consistent with the growing body of research on integrative health services in safety-net settings,^22,32–34^ clinicians in our study reported that IMGVs are providing access to integrative care and peer support, benefiting patients in multiple ways.

Though GMVs and specifically IGMVs are increasingly common, guidelines for billing both public and private insurance for care provided in groups remain unclear.^35,36^ Despite the Affordable Care Act's requirement that health insurance should not discriminate against any licensed provider,^37^ reimbursement is frequently unavailable for licensed non-biomedical providers such as acupuncturists and naturopathic doctors.^38,39^ Given the lack of insurance reimbursement for most complementary health approaches, it is unsurprising that the approaches most commonly used in IGMVs were those that can be offered by biomedical providers with some specialized training or practice (e.g., meditation). Some respondents to our study reported on IGMVs that include other licensed providers such as acupuncturists or chiropractors. However, in our qualitative results, clinicians reported difficulty paying these providers. Broad implementation of IGMV models would be more feasible if both public and private insurers provided reimbursement for a range of licensed health care providers such as naturopathic doctors and acupuncturists. Such reimbursement would not only allow safety-net clinics to hire integrative care providers but also support the infrastructure needed at the clinic and organizational levels to make these programs feasible and sustainable.

Our study found that over 75% of sites with IGMVs used this model to deliver integrative care for chronic pain, and over half provided diabetes care in IGMVs. This is notable given that these and other chronic conditions disproportionately affect low-income individuals, and health care disparities exacerbate this higher prevalence. There is a strong research base for diabetes GMVs,^11,40^ and integrative care may provide additional benefits to patients with diabetes. All authors of this article have ongoing qualitative and mixed-methods projects examining group-based integrative care for chronic pain.^14,26,41^ Our projects suggest that such approaches are a promising innovation that may help reduce or eliminate opioid medication use while allowing organizations to comply with Joint Commission requirements to offer non-pharmacological chronic pain treatment options.^42^ In addition to offering GMVs in which they bill patients' health insurance, most sites offered additional free or low-cost, group-based complementary health approaches such as yoga and tai chi.^34^ Many safety-net clinics have also integrated primary care and mental health care services, and these efforts are visible in the many sites offering group therapy or mental health support groups.^43,44^

### Limitations

This study had a small, targeted sample, limiting the generalizability of the findings. Our sample was 90% female and 83% white, which may not be reflective of the national safety-net workforce providing care in IGMVs. More broadly, there was a potential for bias in favor of GMVs, because we specifically sought out providers currently providing care in GMVs. It is difficult to determine how many organizations are offering IGMVs as clinics rarely advertise these programs on their websites or publish information about them elsewhere. This scoping survey points to the need for additional quantitative and qualitative research on IGMVs as well as broader policy issues of low-income people's access to integrative health care. One example would be a national survey of all federally qualified health centers to assess whether and how they are implementing integrative health care, GMVs, and IGMVs specifically.

We requested that respondents report on IGMVs offered for particular health conditions and using particular treatment approaches, and several commented that our questions about treating specific health conditions (e.g., assuming IGMVs were organized specifically for people with diabetes or chronic pain) did not reflect their programmatic models. Many IGMVs are designed to treat multiple health conditions at once, as is true for integrative health care more broadly. Such an approach is difficult to measure and points to the need for rigorous, mixed-methods approaches to studying integrative health care interventions. A final limitation is that the survey design did not explicitly ask clinicians to name their workplaces, to protect anonymity. We identified cases of multiple survey respondents reporting on the same organizations and removed four respondents from the analysis, but it is possible that other overlaps were missed.

## Conclusion: Implications for Health Equity

Despite these limitations, this study uniquely contributes to our knowledge of IGMVs in safety-net settings by describing the structure and scope of care provided in IGMVs. Though other studies have reported on the outcomes of specific IGMV programs,^15,20,22,23^ to our knowledge, this is the first study of existing IGMV programs across multiple organizations. IGMVs typically provide multidisciplinary care that aims to treat multiple health conditions at once, an approach that is well suited to the needs of safety-net patients and clinicians. Our findings show that despite limited insurance reimbursement for complementary health approaches, safety-net clinicians are creatively increasing access to such treatment by offering it alongside biomedical care in IGMVs. Survey responses indicate that such programs can be used to manage some of the conditions in which major health disparities are present, providing innovative approaches to conditions such as diabetes and chronic pain by increasing access to complementary health approaches. Such care can advance health equity for low-income people receiving care in safety-net settings, including many people of color and immigrants.

## Abbreviations Used

GMV: group medical visit
IGMV: integrative group medical visit
